# Sensory hyperacusis as a predictor of anxiety in adolescence

**DOI:** 10.1111/jcpp.70027

**Published:** 2025-08-13

**Authors:** Foteini Tseliou, Stephan Collishaw, Alice Price, Petroc Sumner

**Affiliations:** ^1^ Division of Psychological Medicine and Clinical Neurosciences, Wolfson Centre for Young People's Mental Health Cardiff University Cardiff UK; ^2^ Division of Psychological Medicine and Clinical Neurosciences, Centre for Neuropsychiatric Genetics and Genomics Cardiff University Cardiff UK; ^3^ School of Psychology Cardiff University Cardiff UK

**Keywords:** Hyperacusis, youth, anxiety, ALSPAC

## Abstract

**Background:**

An increasing number of children report anxiety in early to mid‐adolescence. Early identification of risk during the transition from primary to secondary schools (age 11) could enhance family‐ or school‐based interventions. While known predictors of adolescent anxiety provide some insight, there is a need to identify and understand additional risk factors. Hyperacusis (aversive sensitivity to sound) is correlated with anxiety in children and adults and thus a candidate risk factor longitudinally.

**Methods:**

We explored the predictive potential of auditory hyperacusis using data from the Avon Longitudinal Study of Parents and Children (ALSPAC, *n* = 6,621). Hyperacusis at age 11 was assessed with a single question, while anxiety and related emotional problems were captured by the emotional subscale of the Strength and Difficulties Questionnaire (SDQ‐E) at ages 13, 16 and through longitudinal trajectories (4–16 years).

**Results:**

Hyperacusis significantly predicted anxiety at ages 13 and 16. This predictive effect remained for age 13 even when accounting for pre‐existing anxiety/emotional problems, autism traits and other neurodiversity characteristics (ADHD, dyslexia, dyspraxia). Similar, though less pronounced, patterns emerged at age 16. When testing the four previously identified childhood trajectories of emotional problems, hyperacusis predicted persistent versus decreasing trajectories (the two cases when preschool anxiety is already high) more reliably than increasing from low trajectories (the two cases when preschool anxiety is low). Additional exploratory analyses found that hyperacusis was most strongly associated with SDQ‐E items related to fear, worry, and nervousness; still predicted SDQ‐E scores at age 25, but not adult generalised anxiety disorder, major depressive disorder or suicidal self‐harm.

**Conclusions:**

Taken together, our findings suggest that assessing hyperacusis at age 11 can provide additional predictive insights into the exacerbation and maintenance of anxiety in adolescence.

## Introduction

Anxiety in adolescence is a well‐documented problem and rates grow most rapidly from ages 11 to 17, with more young people affected in recent generations (Armitage, Kwong Alex, et al., 2023). Adolescent anxiety is partially predicted by anxiety at earlier ages, neurodiversity (especially autism and ADHD), and other traits such as behavioural inhibition (e.g. Cabral & Patel, 2020; Fox et al., 2023; Rapee, Creswell, Kendall, Pine, & Waters, 2023), but there is considerable variance in the developmental course of adolescent anxiety which remains unpredictable, hampering early intervention.

For example, Tseliou et al. (2024) identified four trajectories of emotional problems from childhood through adolescence. In one trajectory, anxiety stayed low (67% of individuals). Another showed relatively high anxiety throughout, but this was the smallest group (6%). More common anxiety‐related trajectories demonstrated the difficulty of predicting adolescent anxiety from earlier levels: one trajectory showed high anxiety in adolescence emerging from lower levels in primary school (9%), and the fourth trajectory showed the opposite, where higher anxiety in primary school appeared to naturally resolve in adolescence (18%). Therefore, it is of both practical and theoretical importance to discover further factors that may help predict rising or persistent anxiety in adolescence.

Here, we investigated aversive hypersensitivity to sounds (also known as hyperacusis, sensory discomfort or overresponsivity). Such hyperacusis is quite common (prevalence estimates vary from 3% to >20% depending on definition and method; e.g. Carpenter et al., 2019; Tyler et al., 2014) and correlates with anxiety in children and adults (Ben‐Sasson, Cermak, Orsmond, Carter, & Fogg, 2007; Carpenter et al., 2019; Green & Ben‐Sasson, 2010; Price, Sumner, & Powell, 2021). Hyperacusis is often a core feature of autism (Katikar, Devi, & Prabhu, 2025) and is correlated with other areas of neurodiversity, including ADHD, dyslexia, dyspraxia (Price, Sumner, & Powell, 2025b; Schwarzlose, Tillman, Hoyniak, Luby, & Barch, 2023). Therefore, there is a complex set of relationships between hyperacusis, anxiety, and other factors known to create challenges in adolescence.

The causal mechanisms underlying hyperacusis remain unconfirmed, and likewise the reasons for its correlations with anxiety and neurodiversity dimensions. Sensory sensitivity is sometimes considered a consequence of anxiety, emotional dysregulation or trauma (e.g. Fleming, Harnett, & Ressler, 2024; Green & Ben‐Sasson, 2010). It is also assumed, given its strong association with other autism traits, to be a consequence of the (still largely unknown) brain network differences underlying autism (e.g. He et al., 2021) and possibly other dimensions of neurodiversity (Bijlenga, Tjon‐Ka‐Jie, Schuijers, & Kooij, 2017; Ward et al., 2017; Wilkins, Allen, Gilchrist, & Monger, 2016).

However, there is intriguing evidence that sensory sensitivities might be a risk factor for anxiety, rather than only a consequence of anxiety or associated neurodiversity. In 149 toddlers with autism, sensory sensitivity predicted anxiety a year later (adjusting for initial anxiety), but not vice versa (Green, Ben‐Sasson, Soto, & Carter, 2012). Similarly, preschool sensory sensitivity (age 2–5) predicted anxiety (but not other disorders) at age 6 (*n* = 191; Carpenter et al., 2019). Possibly, sensory sensitivity can be more reliably assessed in very young children than anxiety, but converging results were found in older children: Sensory sensitivity predicted anxiety 1 year later in a large sample (*n* = 10,175) of 9–12‐year‐olds even when controlling for concurrent psychiatric symptoms and autistic traits (Schwarzlose et al., 2023); and visual stress predicted anxiety 3 months later (controlling for loneliness, hopelessness and days of functioning) in 104 12–24‐year‐olds (Hui et al., 2023). As yet, no cohort study has examined a time gap of longer than 1 year or focussed on the important years of mid‐adolescence. If hyperacusis at age 11 helps predict anxiety through adolescence, this could open a research avenue to help better understand risk factors and causal chains for (aspects of) adolescent anxiety.

### Aims of the study

Using a UK population cohort, we aimed to assess whether a single question about hyperacusis at age 11 helps to predict anxiety and related emotional problems in adolescence (ages 13 and 16) and relatedly, whether it can distinguish previously identified developmental trajectories of anxiety, given known anxiety levels in primary school (i.e. persistent relative to naturally resolving anxiety, and adolescent‐increasing anxiety relative to staying low).

At the same time, we ask to what extent any longitudinal prediction of later anxiety is accounted for by pre‐existing anxiety/emotional problems and neurodiversity characteristics (autism, ADHD, dyslexia and dyspraxia). In further exploratory analyses, we assessed which questions about emotional problems drove any association with prior hyperacusis, whether hyperacusis at age 11 can predict adult mental health outcomes, and whether hyperacusis moderates the known association between childhood neurodiversity and adolescent anxiety.

## Methods

### Study participants

We used data from the Avon Longitudinal Study of Parents and Children (ALSPAC), an ongoing longitudinal population‐based UK cohort (Boyd et al., 2013). ALSPAC enrolled 14,541 women residing in the Avon area, who had an expected delivery date between 1st April 1991 and 31st December 1992 (Fraser et al., 2013). There were 14,062 live births, and 13,988 children reached 1 year. In 1998, the sample was bolstered with 913 eligible families who had not originally joined the study (Northstone et al., 2019). Details of all data collected are available at the study website (http://www.bristol.ac.uk/alspac/researchers/our‐data/). Sex was assessed via maternal report at birth. Data gathered from participants aged 22 onwards used Research Electronic Data Capture (REDCap) hosted at the University of Bristol (Harris et al., 2009, 2019).

Our analysis sample comprised 6,621 individuals (50.8% female, 95.3% white) with data on hyperacusis at age 11 and at least one of the outcomes of interest: emotional problems at 13 and 16, anxiety, depression and self‐harm at 24, and emotional problems at 25. For twins, the second‐born twin was excluded to avoid non‐independent observations.

Ethical approval for the study was obtained from the ALSPAC Ethics and Law Committee and the Local Research Ethics Committees. Informed consent for the use of data collected via questionnaires and clinics was obtained from participants following the recommendations of the ALSPAC Ethics and Law Committee at the time.

### Hyperacusis

At age 11, children attended a hearing assessment during which a hyperacusis and tinnitus interview was also carried out. Hyperacusis was defined as oversensitivity or distress to particular everyday sounds rather than to loud sounds, with the question phrased ‘ever experience oversensitivity or distress to particular sounds?’ (Hall, Humphriss, Baguley, Parker, & Steer, 2016). This single question was our measure of hyperacusis (see ‘Limitations’ section for discussion). Those that answered yes were further questioned on wearing ear protection, avoiding places or activities, estimated length of time child had experienced hyperacusis, and oversensitivity to light/colours, touch, pain, smell or taste. Thus, sensitivity to other senses was not assessed independently of hyperacusis to sounds, but only as sub‐questions if hyperacusis to sounds was reported. Small N meant that we could not meaningfully analyse the sub‐questions.

### Outcome measure: SDQ‐E

The emotional symptoms subscale of the Strength and Difficulties Questionnaire (SDQ), a brief validated screening tool for common mental health problems in children and young people (Armitage, Kwong Alex, et al., 2023; Goodman, 1999), was completed by the child's main carer (usually the child's mother) when children were approximately 4, 7, 8, 9, 11, 13 and 16/17 years old. The emotional symptoms scale (SDQ‐E) contains five items, including questions about fears, anxiety, depressed mood, nervousness and somatic complaints, each rated on a 3‐point scale (2 = *Certainly true*; 1 = *Sometimes true*; 0 = *Not true*; scale range 0–10). We used these SDQ‐E scores at age 13 and 16 (internal consistency: *α* = .67 at 13 years, *α* = .71 at 16 years; Armitage, Tseliou, et al., 2023) as our main outcomes.

The SDQ‐E has been validated as a measure of emotional disorders for individuals aged up to 18 years (Armitage, Tseliou, et al., 2023; Riglin et al., 2024) and shown high accuracy for discriminating cases of generalised anxiety (AUC = 0.80–0.93) or any anxiety disorders (AUC = 0.74–0.83; Armitage, Tseliou, et al., 2023). Subsets of items have been examined separately, such as worry to represent generalised anxiety or low mood to represent depression (e.g. Armitage, Tseliou, et al., 2023; Tseliou et al., 2024), but the items correlate with each other and the overall score generally performs better than individual items at discriminating anxiety disorders (Armitage, Tseliou, et al., 2023), so we simply used the overall score here.

Previously identified trajectories of anxiety‐related emotional problems (Tseliou et al., 2024) based on SDQ‐E scores across all the timepoints (4–16/17 years) were also used. These were previously chosen based on best model fit indices (including loglikelihood (LL), sample size adjusted Bayesian information criterion (BIC) and entropy) and implemented using a bias‐adjusted three‐step approach. The solution that best fitted the data included a class with persistently low emotional problems, one with decreasing levels of emotional problems, one with increasing levels of emotional problems, and a fourth class of persistent high levels of emotional problems throughout childhood and adolescence.

### Covariates

We included childhood covariates to test the extent to which any predictive effect of hyperacusis was accounted for by contemporary emotional problems or neurodivergence. As a proxy for autism spectrum disorders, social communication difficulties were assessed at 11 years using the Social and Communication Disorders Checklist (SCDC) (Skuse et al., 2009), with the cut‐point set at > = 9. For ADHD symptoms, we used the hyperactivity/inattention subscale of the Strength and Difficulties Questionnaire (SDQ‐H) at age 11. The SDQ‐H contains five items, including questions about restlessness, fidgeting, lack of concentration, thinking before acting and finishing work, each rated on a 3‐point scale (2 = *Certainly true*; 1 = *Sometimes true*; 0 = *Not true*; range 0–10). The presence of dyslexia and dyspraxia were assessed via parent report (‘Have you ever been told that your child has dyslexia/ dyspraxia?’) at 10 years.

### Mental health measures in young adulthood

For secondary analysis exploring the predictive power of hyperacusis beyond adolescence, we used outcome measures of mental health at ages 24 and 25. The Clinical Interview Schedule–Revised (CISR) (Lewis, Pelosi, Araya, & Dunn, 1992) was used to assign DSM‐IV diagnoses of generalised anxiety disorder (GAD) and major depressive disorder (MDD) at 24 years. Lifetime suicidal self‐harm was assessed by self‐report at age 24. The SDQ emotional symptoms scale (SDQ‐E) was also self‐completed at age 25 (range 0–10).

### Sense checks and cohort characteristics

Hyperacusis was also measured at age 28 in the cohort as part of the Sussex Screener for Misophonia (SSfM) (Rinaldi & Simner, 2025). We checked whether this correlated with hyperacusis at age 11.

Since hyperacusis is expected to correlate with contemporary anxiety, autism traits and other aspects of neurodiversity, we tested these associations for this cohort using the SDQ‐E score at age 11 and the proxies for autism traits, ADHD symptoms, dyslexia and dyspraxia described under covariates above.

To further characterise our sample, we also used key parental, family and individual data that have previously been related to emotional problems (e.g. Tseliou et al., 2024). Maternal anxiety was measured at 2 years via the self‐report question ‘had anxiety or nerves’ with available responses ranging from no, ‘yes, did not see doctor’ and ‘yes & saw doctor’ (with the two latter grouped together as yes). Family poverty was assessed when the child was aged 11 using household income of less than 60% of the median for the sample (<£240 per week) (Weavers et al., 2021). Household crowding index was defined as the total number of residents in the household excluding the newborn infant (accounting for the total number of rooms) (Melki, Beydoun, Khogali, Tamim, & Yunis, 2004) at 3 years.

### Analysis plan

#### Sense checks and cohort characteristics

Descriptive statistics, percentages for binary and means (standard deviation) for continuous variables of interest, were used to provide key characteristics of our cohort. Odds ratios were used for sense checks of expected associations (hyperacusis at age 28, and contemporary emotional problems and neurodevelopmental problems), as well as for other cohort characteristics for completeness.

#### Main analyses: Does hyperacusis predict emotional problems and distinguish trajectories

Negative binomial models were used to assess whether hyperacusis at age 11 predicts SDQ‐E scores at ages 13 and 16, and to what extent this is accounted for by pre‐existing SDQ‐E scores and neurodiversity measures. Model 1 included only hyperacusis as a predictor, Model 2 added SDQ‐E scores at age 11, Model 3 also added autism traits, and Model 4 further added ADHD symptoms, as well as dyslexia and dyspraxia (reported diagnosis).

To assess the related question of whether hyperacusis can help distinguish the previously identified SDQ‐E trajectories (Tseliou et al., 2024), binary logistic regression models were used to compare increasing from low and persistent from decreasing.

We imputed the covariates and outcomes to account for missingness in these variables (see Table S1). Multiple imputation using Fully Conditional Specification was used in IBM SPSS Statistics Version 29. In total, 50 imputations (10 iterations in each instance) were implemented. See Table S1 for proportions of missing data in each measure.

#### Secondary analyses

To explore which of the five items in the SDQ‐E were associated with hyperacusis, we ran separate analyses with hyperacusis predicting each of the five symptoms.

To explore longer‐term associations of hyperacusis with adult mental health outcomes, we used logistic regression and negative binomial models with outcome measures of GAD, MDD, self‐harm at age 24, and SDQ‐E at 25. Model 1 used only hyperacusis as a predictor; Model 2 added SDQ‐E scores at age 11, Model 3 also added autism traits, and Model 4 further added ADHD symptoms, dyslexia, and dyspraxia (reported diagnosis).

To explore whether hyperacusis moderates the association of childhood neurodivergence with adolescent anxiety, separate models were run to test if any associations between adolescent SDQ‐E and neurodiversity proxies at 11 were moderated by hyperacusis.

### Role of the funding source

The funders of the study had no role in the study design, data collection, data analysis, data interpretation, or writing of the report.

## Results

### Sample characteristics

In our sample of 6,621 children who attended the hearing clinic, 3.7% reported hyperacusis, with 38.4% of these children being female. Sense checks showed that these hyperacusis reports were correlated with adult hyperacusis (age 28, OR 3.01, 95% CI: 1.05, 8.64), and also broadly associated with expected characteristics of childhood neurodiversity and emotional problems (Table 1). Interestingly, reports among females were lower than males, which we return to in discussion.

**Table 1:** Cohort characteristics and data sense checks

	No hyperacusis (*N* = 6,379; 96.3%)	Hyperacusis (*N* = 242; 3.7%)	Odds ratio (95% CI)
Individual			
Female	51.2%	38.4%	0.59 (0.46, 0.77)
Emotional problems at 11*	1.51 (1.69)	1.85 (1.92)	1.11 (1.03, 1.19)
Autism traits at 11	4.9%	12.8%	2.83 (1.91, 4.19)
ADHD symptoms at 11*	2.91 (2.18)	3.12 (2.48)	1.04 (0.98, 1.11)
Dyslexia at 10	8.2%	13.1%	1.72 (1.04, 2.83)
Dyspraxia at 10	9.0%	13.8%	1.67 (0.87, 3.23)
Family			
Maternal anxiety	18.3%	22.4%	1.29 (0.93, 1.78)
Household crowding at 3*	2.30 (0.85)	2.20 (0.84)	0.86 (0.73, 1.01)
Family poverty at 11	31.9%	33.1%	0.95 (0.71, 1.26)

Data represent percentages for binary and means (standard deviation) for continuous variables (marked with *) in the imputed dataset.

### Does hyperacusis predict adolescent anxiety?

**Figure 1:**
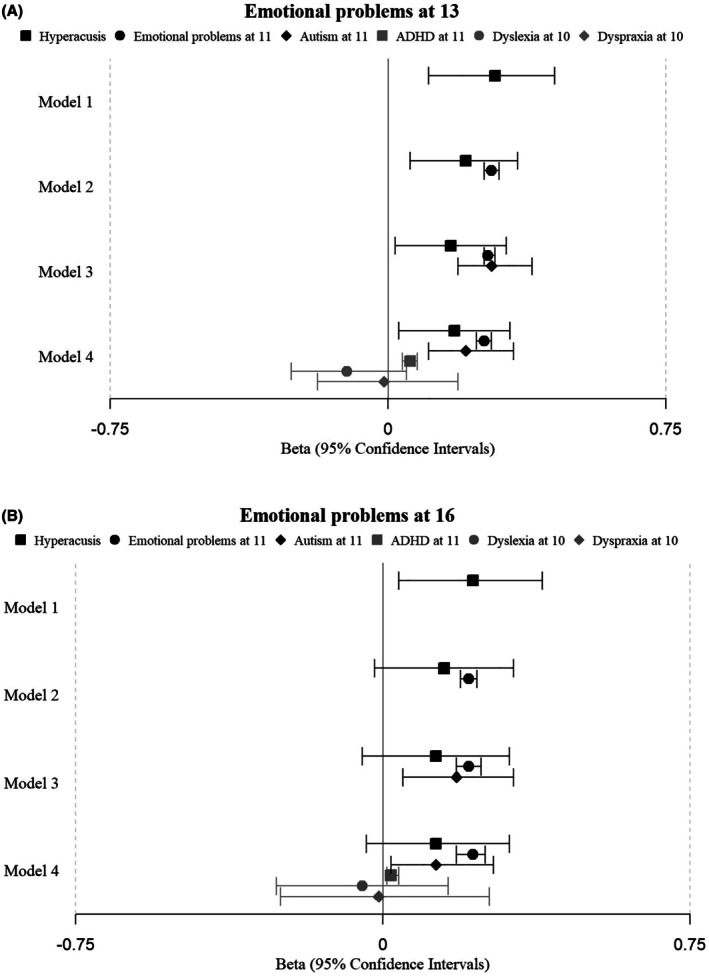
Regression models of the predictive value of hyperacusis at 11 for emotional problems at 13 and 16 in the imputed dataset. Model 1 included only hyperacusis as a predictor. Models 2–4 added further age 10/11 predictors: emotional problems, autism, ADHD, dyslexia and dyspraxia

Figure 1 shows the outcome of regression models assessing our main question: whether hyperacusis at 11 helps predict SDQ‐E scores in adolescence, and to what extent any predictive effect of hyperacusis is already accounted for by pre‐existing emotional problems and neurodiversity characteristics. Hyperacusis significantly predicted SDQ‐E scores at age 13 (Figure 1A) even when adjusting for SDQ‐E at 11 (Model 2), as well as after additional adjustment for autism traits (Model 3), ADHD symptoms, dyslexia and dyspraxia (Model 4). Therefore, hyperacusis at age 11 is useful additional information for assessing risk for adolescent anxiety.

A broadly similar pattern is evident for age 16 (Figure 1B), but with more uncertainty, so that the confidence intervals for hyperacusis start to encompass zero when additionally adjusting for SDQ‐E at age 11 and neurodiversity traits (Models 2, 3 and 4). This likely reflects the increased noise and missingness in the data for age 16 (Table S1).

Next, we tested associations of hyperacusis with anxiety trajectories, focusing on two comparisons: (1) comparing two trajectory groups with low anxiety symptoms in childhood, does hyperacusis distinguish those with a rising trajectory of anxiety (relative to those who stayed low)?; (2) comparing two groups with elevated anxiety symptoms in primary school, does hyperacusis help distinguish those with persistent anxiety from those showing a reduction of symptoms during adolescence? (Figure S1) Hyperacusis appears to show better prediction for the latter comparison when adjusting for childhood neurodevelopmental covariates (Table 2). Therefore, it seems assessing hyperacusis is especially useful for children who are already showing elevated anxiety.

**Table 2:** Logistic regressions of the association of hyperacusis with increasing versus low (reference) and persistent versus decreasing (reference) trajectory classes of emotional problems

	Increasing vs. Low (ref)	Persistent vs. Decreasing (ref)
Unadjusted		
Hyperacusis	1.61 (1.05, 2.47)	2.31 (1.39, 3.84)
Adjusted for autism at 11		
Hyperacusis	1.33 (0.86, 2.08)	2.14 (1.27, 3.62)
Autism traits at 11	5.08 (3.71, 6.95)	3.68 (2.56, 5.28)
Adjusted for autism and other neurodevelopmental difficulties at 11		
Hyperacusis	1.25 (0.74, 2.13)	2.04 (1.13, 3.71)
Autism traits at 11	2.65 (1.79, 3.90)	2.92 (1.84, 4.62)
ADHD symptoms at 11*	1.25 (1.20, 1.31)	1.12 (1.05, 1.19)
Dyslexia at 10	0.74 (0.34, 1.58)	1.72 (0.82, 3.57)
Dyspraxia at 10	1.39 (0.48, 4.02)	0.83 (0.35, 2.00)

Data show odds ratios and 95% confidence intervals in the imputed dataset.

### Further analyses

#### Which adolescent emotional symptoms does hyperacusis predict?

The association with hyperacusis was strongest and more persistent for the questions most directly related to anxiety (worry, nervousness and fears; Table 3), consistent with a stronger association with anxiety than depression in cross‐sectional studies (Jüris, Gerhard, Christian, & Ekselius, 2013).

**Table 3:** Regression models of the association of hyperacusis and symptoms of emotional problems at 13 and 16

	Age 13	Age 16
Somatic complaints	.15 (0.01, 0.29)	.01 (−0.15, 0.17)
Worry	.24 (0.10, 0.39)	.22 (0.07, 0.38)
Depressed mood	.15 (0.01, 0.29)	.11 (−0.05, 0.27)
Nervousness	.15 (0.01, 0.29)	.17 (0.02, 0.33)
Fears	.27 (0.12, 0.41)	.19 (0.03, 0.35)

Data represent beta coefficient and 95% CI in the imputed dataset.

#### Does the predictive value of hyperacusis extend to adult outcomes?

ALSPAC data also contain data at age 24 for DSM‐IV defined diagnoses of generalised anxiety disorder (GAD), major depressive disorder (MDD) and self‐harm, as well as a further measure of emotional problems (SDQ‐E) at age 25. The predictive effect of hyperacusis at age 11 was in the expected direction for GAD and adult emotional problems, but only significant for the SDQ measure unadjusted for age‐11 emotion problems. Due to high missingness in the adult data, we repeated the analysis for non‐imputed data to check for consistency. We found similar results (numerically larger effects, but same pattern of significance; Table S2). Thus, the longitudinal association of childhood hyperacusis with anxiety traits appears to extend into adulthood, but the data available are suboptimal (Table 4).

**Table 4:** Univariate logistic regression models of the association of hyperacusis at 11 and outcomes in young adulthood

Outcomes	Model 1 OR (95% CI)	Model 2 OR (95% CI)	Model 3 OR (95% CI)	Model 4 OR (95% CI)
GAD (CISR) at 24	1.50 (0.90, 2.52)	1.44 (0.86, 2.43)	1.41 (0.84, 2.37)	1.40 (0.83, 2.34)
MDD (CISR) at 24	1.03 (0.60, 1.75)	1.00 (0.59, 1.71)	0.97 (0.57, 1.66)	0.98 (0.57, 1.69)
Self‐harm at 24	1.05 (0.63, 1.75)	1.02 (0.62, 1.68)	1.01 (0.62, 1.66)	1.02 (0.62, 1.69)

	Model 1 beta (95% CI)	Model 2 beta (95% CI)	Model 3 beta (95% CI)	Model 4 beta (95% CI)
Emotional problems at 25*	.20 (0.02, 0.38)	.13 (−0.05, 0.30)	.10 (−0.08, 0.28)	.10 (−0.07, 0.28)

Data represent odds ratios and 95% CI for binary variables and beta coefficient and 95% CI for continuous variables in the imputed dataset.

#### Moderation of conversion of childhood neurodivergence to anxiety

It is known that childhood neurodivergence can be associated with adolescent anxiety (Zaboski & Storch, 2018), but it is not fully known why anxiety develops in some but not all neurodivergent children. We therefore asked if this association in the ALSPAC data is moderated by hyperacusis. However, upon using hyperacusis as an interaction term in univariate regression models of childhood measures and emotional problems at age 13 and 16, there were no significant effects (Table 5).

**Table 5:** Univariate regression models of the association of childhood neurodivergence and emotional problems at 13 and 16, testing potential moderation by hyperacusis

	Main effect hyperacusis	Main effect covariate	Interaction (Age 13)	Main effect hyperacusis	Main effect covariate	Interaction (Age 16)
Autism traits at 11	.18 (0.01, 0.37)	.62 (0.49, 0.76)	.06 (−0.38, 0.50)	.15 (−0.04, 0.35)	.44 (0.29, 0.59)	.11 (−0.35, 0.57)
ADHD symptoms at 11*	.30 (−0.03, 0.56)	.12 (0.10, 0.13)	.01 (−0.08, 0.05)	.22 (−0.06, 0.50)	.07 (0.04, 0.08)	.01 (−0.07, 0.07)
Dyslexia at 10	.21 (0.03, 0.40)	.16 (0.01, 0.32)	.33 (−0.18, 0.87)	.21 (0.02, 0.40)	.12 (−0.06, 0.29)	.04 (−0.47, 0.55)
Dyspraxia at 10	.24 (0.06, 0.42)	.21 (0.02, 0.40)	.21 (−0.37, 0.79)	.23 (0.05, 0.42)	.12 (−0.08, 0.34)	−.14 (−0.71, 0.43)

Data represent beta coefficient and 95% CI in the imputed dataset (* denotes continuous variable).

## Discussion

We found that a single hyperacusis question for children at 11 years old (‘*have you ever experienced oversensitivity or distress to particular sounds’*) robustly predicted anxiety (worry, nervousness, fears) at ages 13 and 16 years. For the smaller age gap, this predictive effect was additional to that already supplied by anxiety and neurodiversity measures at age 11. In terms of childhood‐to‐adolescence trajectories, hyperacusis also distinguished those with high previous anxiety who showed persistent anxiety relative to those whose anxiety improved over adolescence.

We speculate that such prediction could be enhanced with a broader assessment of sensory discomfort across the senses. Auditory sensitivity is the most commonly reported in adults (Price, 2023), but it is closely followed by visual and tactile sensitivities, and in young children, tactile sensitivities have been reported as most common (Carpenter et al., 2019). While there is correlation across the senses (Powell et al., 2020), many children experiencing sensory discomfort would be missed by assessing a single sense. We were limited by the existing data to using only auditory hyperacusis because hypersensitivities in other senses were not independently measured. This is both a weakness and a potential strength. Ideally, different triggers across multiple senses would be assessed, but for practical purposes, if one simple‐to‐administer question has predictive power, this allows pragmatic application and could imply that fuller assessment, where possible, might provide improved prediction.

Moreover, there are subfactors of sensitivity within senses (Price, Sumner, & Powell, 2025a). Within audition, distinctions have been drawn between loudness hyperacusis, other kinds of hyperacusis and misophonia (an emotive reaction to eating and breathing sounds) (Jastreboff & Hazell, 2008). In the case of visual sensitivities, where this is most explored, the subfactors are transdiagnostic—they do not neatly map onto different psychiatric conditions and areas of neurodiversity—but there are relative differences in their association with anxiety and other conditions. Therefore, we speculate that the predictive effect for a single question may be a lower bound for what is possible with enhanced (though slightly more time‐consuming) assessment of different factors.

### Hyperacusis as a risk factor for anxiety?

One interpretation of the results is that hyperacusis acts as a direct risk factor for exacerbating or maintaining anxiety. What hypothetical causal pathways could be present? At a descriptive level, people who experience both hyperacusis and anxiety often perceive a direct link between the two, as shown in qualitative reports (e.g. ‘*I had a noisy job where I basically had panic attacks in the supply closet every day as a result of all the noise’; ‘Everything feels louder and brighter when I start getting overstimulated. Crowds can be very hard… My heart rate increases, my face gets hot, I get sweaty, and my anxiety increases…’*; Price, 2023). This implies that sensory sensitivity can make everyday experiences overwhelming and anxiety inducing, especially in environments where aversive stimuli might be unpredictable (Grillon et al., 2008).

More formally, context conditioning could be a mechanism: If an unconditioned stimulus (e.g. aversive sound) occurs unpredictably within a context, then the fear response can broaden to the entire context, such as school or social situations (Pfeiffer, Brusilovskiy, Bauer, & Salzer, 2014). The avoidance, hypervigilance, and corresponding physiological arousal resulting from aversive sensory reactions could lead to generalised symptoms of anxiety (Green & Ben‐Sasson, 2010). This is related to the theory of interoceptive fear conditioning, whereby internal sensations (in this case the experience of sensory discomfort) are paired with fear and then come to elicit fear more generally (e.g. Bouton, Mineka, & Barlow, 2001). The idea also aligns with Hofmann's model of mood disorders (Hofmann, Sawyer, Fang, & Asnaani, 2012), whereby emotion regulation strategies (i.e. avoidance) contribute to the development of anxiety, and are supported by mediation analyses (McMahon, Anand, Morris‐Jones, & Rosenthal, 2019). Other cognitive traits may contribute to maintaining these relationships, for example intolerance of uncertainty (Panchyshyn, Tekok‐Kilic, Frijters, & Tardif‐Williams, 2023; Uljarević, Lane, Kelly, & Leekam, 2016), and behaviourally inhibited temperament (Fox et al., 2023).

Hypervigilance, once elicited, can potentially create a problematic feedback cycle. Investigations of attention bias suggest that anxious individuals have increased hypervigilance to their environment, and subsequent difficulty disengaging their attention from threat‐relevant stimuli (Mobini & Grant, 2007; Pergamin‐Hight, Pine, Fox, & Bar‐Haim, 2016). Hypervigilance could in turn increase the sensory response to aversive stimuli, exacerbating the discomfort experienced.

If hyperacusis is a risk factor for exacerbating or maintaining anxiety, interventions to reduce the prevalence of aversive stimuli in school environments, or therapies to help reduce aversive responses to those stimuli, may help ameliorate the exacerbation of adolescent anxiety (Muskett, Radtke, White, & Ollendick, 2019).

### Hyperacusis as a marker for increased anxiety risk?

A second interpretation is that hyperacusis may have a reliable correlation with some unmeasured factor that raises anxiety risk. What might hyperacusis be a marker for? One possibility is migraine, which has correlations with both anxiety and sensory sensitivities across children, adolescents, and adults (Falla et al., 2022; Price et al., 2021). Migraine was not available in our cohort, but the SDQ‐E contains a ‘somatic’ question (‘complains of headache/stomach‐ache’), which likely correlates with migraine. Yet this question did not drive the relationship with hyperacusis (Table 3), so it seems hyperacusis was not simply marking migraine.

A more general hypothesis is that sensory hypersensitivity is a marker for cortical excitability and may reflect an aspect of neurodiversity that current understanding and classifications are poor at capturing. Intriguingly, sensory hypersensitivity has shown associations with synaesthesia as well as a host of psychiatric, neurodevelopmental and neurological conditions (Price, 2023; Ward et al., 2017). The main theory relates to excitation/inhibition balance in cortical circuits. Visual discomfort has been related to greater responsivity in the visual cortex (Ward, 2019; Wilkins, 2021), while tactile aversion has been related to lower GABA concentration in the sensorimotor cortex (He et al., 2021). If the balance of excitation/inhibition is altered more generally across brain networks, this could underlie other symptoms of anxiety. For example, the amygdala has been associated both with differential activation in anxiety disorders (Shin & Liberzon, 2010) and with distinct patterns of connectivity in sensory discomfort (Schwarzlose et al., 2023). Similarly, GABAergic systems are a therapeutic target in anxiety (Farach et al., 2012; Möhler, 2012) and are also implicated in heightened sensory sensitivity (Edden, Muthukumaraswamy, Freeman, & Singh, 2009; He et al., 2021; Orekhova et al., 2019; Stroganova et al., 2015). However, theories of excitation/inhibition have been posited for many conditions and areas of neurodiversity, from schizophrenia to migraine (Howes & Shatalina, 2022; Nguyen, McKendrick, & Vingrys, 2016), and as yet these theories lack the specificity to explain the multitude of different symptom presentations.

Since it remains unknown what causes hyperacusis (there are likely several causes in the peripheral as well as central auditory system; Pienkowski et al., 2014), it remains speculative as to why hyperacusis is connected to anxiety. One study (Hall et al., 2016) used the same ALSPAC cohort to assess early life and auditory risk factors for hyperacusis. They replicated the known associations with tinnitus and autism diagnosis, and found smaller associations with maternal education, hospitalisation after birth, and a tendency for larger transient otoacoustic emissions. The latter likely reflects an inner ear cause for some children (possibly cochlear gain, which may also relate to tinnitus), and neonatal illness is a known risk for auditory neurodevelopment (Hall et al., 2016). Maternal education is discussed under limitations below.

### Limitations

ALSPAC, like other prospective birth cohorts, experienced non‐trivial participant dropout over time (missing SDQ data of 20%, 34% and >50% for ages 13, 16 and 24 in our study, see Table S1). Such dropout often correlates with increased risk for mental health problems and family disadvantage (Tejerina‐Arreal et al., 2020). Previous sensitivity analyses suggest this did not affect SDQ‐E validity (Armitage, Tseliou, et al., 2023), but it will have limited the sample size and estimated prevalence of hyperacusis; indeed around two thirds of diagnosed autistic children did not attend the age 11 research clinic (Williams, Thomas, Sidebotham, & Emond, 2008), and many of these children would have hyperacusis and anxiety.

While many prevalence estimates for sensory sensitivity are not specific to one sense, 3.7% is low even for auditory alone (e.g. Tyler et al., 2014). In this cohort, hyperacusis was assessed as part of a hearing and tinnitus clinic and, in addition to attendance biases, the setting may have primed responders to think in terms of hearing problems rather than wider auditory experience, thus implicitly imposing a higher threshold on answering yes than in questionnaire research. It is noteworthy that hyperacusis correlates with higher mother's educational status in this cohort, which implies either selection bias in attending the clinic or a response bias due to awareness of the hyperacusis questions. Previous research reported an apparently opposite correlation: sensory overresponsivity (mainly tactile) correlated with poverty (Carpenter et al., 2019), although family poverty markers were not associated with hyperacusis in ALSPAC (see Table 1 and Hall et al., 2016). Other than reducing N, and therefore effect sizes, it is not obvious how the dropout or biases would have affected the predictive effect of hyperacusis for adolescent anxiety.

The limitation associated with asking a single hyperacusis question about a single sense has been discussed above. This simplicity can also be considered a pragmatic strength, given that one question still informed prediction. A further limitation relates to the assessment of anxiety, which here used the SDQ‐E parent‐reported symptom screen. Although this has reasonable validity as a general index of anxiety in children and young people, it was not possible to distinguish between different forms of anxiety (e.g. separation anxiety, social anxiety, generalised anxiety or panic), which show distinct patterns of ages of onset and developmental course. Lastly, autism traits and ADHD symptoms were assessed with questionnaires associated with these conditions rather than with formal diagnosis or a full assessment of all symptom dimensions.

## Conclusion

This longitudinal study highlights the predictive role of childhood hyperacusis in adolescent anxiety, suggesting that sensory sensitivity may either exacerbate anxiety or act as a marker for neurodevelopmental differences linked to the onset and persistence of anxiety. Future research should explore the potential of enhanced sensory assessments to improve the prediction of risk beyond auditory sensitivity alone.

## Ethics statement

Ethical approval for the study was obtained from the ALSPAC Ethics and Law Committee and the Local Research Ethics Committees. ALSPAC was initially approved by the Bristol and Weston Health Authority (E1808 Children of the Nineties: Avon Longitudinal Study of Pregnancy and Childhood (ALSPAC). (28th November 1989)), the Southmead Health Authority (49/89 Children of the Nineties—‘ALSPAC’. (5th April 1990)) and the Frenchay Health Authority (90/8 Children of the Nineties. (28th June 1990)). The hyperacusis variable was collected at the age 11 clinic, which was approved by the following committees: Southmead (North Bristol Trust) (137/02: ALSPAC—Focus at 11+. (17th April 2003), United Bristol Healthcare Trust (E5691 ALSPAC Focus @ 11+ Reciprocal) (29th May 2003)), Frenchay (North Bristol Trust) (2002/110 Avon Longitudinal Study of Parents and Children (ALSPAC) Assessments at Age Eleven Plus. (23rd June 2003)) and Weston Area Health Trust (E325 (R)—137/02 Avon Longitudinal Study of Parents and Children (ALSPAC) Assessments at Age Eleven Plus (28th February 2003)). Informed consent for the use of data collected via questionnaires and clinics was obtained from participants following the recommendations of the ALSPAC Ethics and Law Committee at the time. No further ethics approval was required for the analyses in this manuscript.

**Key points**

- What is known: Anxiety correlates with hyperacusis in many cross‐sectional studies, but whether anxiety precedes hyperacusis or anxiety precedes anxiety is debated.
- What is new: Hyperacusis at age 11 helps predict anxiety at age 13 and 16 even when adjusting for pre‐existing anxiety at age 11.
- What is relevant: Measuring hyperacusis at the transition from primary to secondary education could enhance early mental health risk detection.

## Supporting information


**Table S1.** Proportion of missing data in key factors in the sample (*n* = 6,621) before imputation.
**Figure S1.** ROC curve comparing low versus increasing and decreasing versus persistent trajectories in the imputed dataset using the fully adjusted model from Table 2.
**Table S2.** Univariable logistic regression models of the association of hyperacusis at 11 and outcomes in young adulthood.

## Data Availability

Scripts used for the analyses conducted in this study are available on request from the corresponding author, FT. The data are available upon request and subject to cohort‐specific executive data access procedures. ALSPAC data access is regulated through a system of managed open access. Please note that the ALSPAC study website contains details of all the data that is available through a fully searchable data dictionary and variable search tool (http://www.bristol.ac.uk/alspac/researchers/our‐data/).
