# Supplementary material for: Sensory hyperacusis as a predictor of anxiety in adolescence
Source: J Child Psychol Psychiatry. 2025 Aug 13;67(5):641–51. doi: 10.1111/jcpp.70027 (PMC13102050; doi:10.1111/jcpp.70027)
Supplement: Supplementary file 1 — Table S1. Proportion of missing data in key factors in the sample (n = 6,621) before imputation. Figure S1. ROC curve comparing low versus increasing and decreasing versus persistent trajectories in the imputed dataset using the fully adjusted model from Table 2. Table S2. Univariable logistic regression models of the association of hyperacusis at 11 and outcomes in young adulthood. [file JCPP-67-641-s001.docx]

**Supplementary material**

Table S1. Proportion of missing data in key factors in the sample (n =6621) before imputation

|  | **% Missing** |
| --- | --- |
| **Individual** |  |
| Hyperacusis | 0% |
| Female | 0% |
| Emotional problems at 11 | 15.1% |
| Autism traits at 11 | 16.8% |
| ADHD symptoms at 11 | 15.1% |
| Dyslexia at 10 | 15.8% |
| Dyspraxia at 10 | 17.2% |
| **Family** |  |
| Maternal anxiety | 11.4% |
| Household crowding at 3 | 14.1% |
| Family poverty at 11 | 20.8% |
| **Outcomes** |  |
| Emotional problems at 13 | 20.3% |
| Emotional problems at 16 | 34.2% |
| GAD (CISR) at 24 | 53.9% |
| MDD (CISR) at 24 | 53.8% |
| Self-harm at 24 | 53.8% |
| Emotional problems at 25 | 48.5% |

Figure S1. ROC curve comparing low versus increasing and decreasing versus persistent trajectories in the imputed dataset using the fully adjusted model from Table 2.


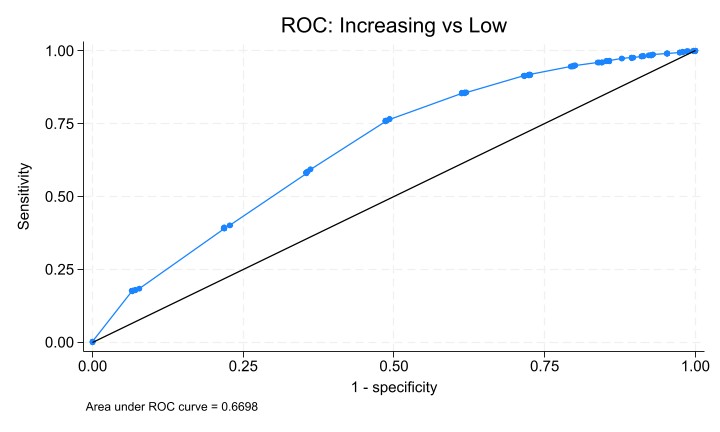

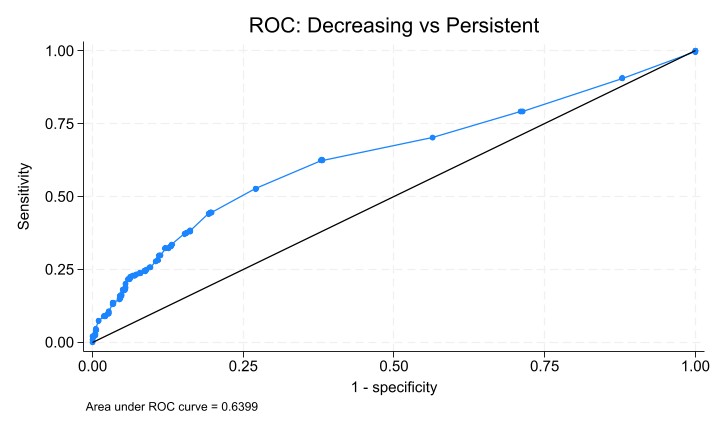


Table S2. Univariable logistic regression models of the association of hyperacusis at 11 and outcomes in young adulthood. Data represent Odds ratios and 95%CI for binary variables and Beta coefficient and 95%CI for continuous variables in the non-imputed dataset

| Outcomes | Model 1 OR (95%CI) | Model 2 OR (95%CI) | Model 3 OR (95%CI) | Model 4 OR (95%CI) |
| --- | --- | --- | --- | --- |
| GAD (CISR) at 24 | 1.67 (0.39 2.14) | 1.71 (0.39 2.28) | 1.72 (0.40 2.32) | 1.69 (0.38 2.26) |
| MDD (CISR) at 24 | 0.99 (0.55 1.78) | 1.09 (0.56 2.12) | 1.13 (0.58 2.19) | 1.05 (0.54 2.05) |
| Self-harm at 24 | 1.06 (0.67 1.66) | 1.08 (0.67 1.76) | 1.08 (0.66 1.75) | 1.05 (0.64 1.71) |
|  | Model 1 beta (95%CI) | Model 2 beta (95%CI) | Model 3 beta (95%CI) | Model 4 beta (95%CI) |
| Emotional problems at 25 * | **0.28 (0.07 0.51)** | 0.15 (-0.06 0.37) | 0.12 (-0.10 0.33) | 0.12 (-0.06 0.37) |

*For beta coefficient, a *β* > 0 means a positive effect, while a *β* < 0 means a negative effect
